# Heat Shock Factor 1 Contributes to Ischemia-Induced Angiogenesis by Regulating the Mobilization and Recruitment of Bone Marrow Stem/Progenitor Cells

**DOI:** 10.1371/journal.pone.0037934

**Published:** 2012-05-24

**Authors:** Masayuki Kubo, Tao-Sheng Li, Hiroshi Kurazumi, Yoshihiro Takemoto, Mako Ohshima, Yumi Yamamoto, Arata Nishimoto, Akihito Mikamo, Mitsuaki Fujimoto, Akira Nakai, Kimikazu Hamano

**Affiliations:** 1 Department of Surgery and Clinical Science, Yamaguchi University Graduate School of Medicine, Ube, Yamaguchi, Japan; 2 Department of Stem Cell Biology, Nagasaki University Graduate School of Biomedical Science, Nagasaki, Nagasaki, Japan; 3 Department of Biochemistry and Molecular Biology, Yamaguchi University Graduate School of Medicine, Ube, Yamaguchi, Japan; University of Illinois at Chicago, United States of America

## Abstract

Bone marrow (BM)-derived stem/progenitor cells play an important role in ischemia-induced angiogenesis in cardiovascular diseases. Heat shock factor 1 (HSF1) is known to be induced in response to hypoxia and ischemia. We examined whether HSF1 contributes to ischemia-induced angiogenesis through the mobilization and recruitment of BM-derived stem/progenitor cells using HSF1-knockout (KO) mice. After the induction of ischemia, blood flow and microvessel density in the ischemic hindlimb were significantly lower in the HSF1-KO mice than in the wild-type (WT) mice. The mobilization of BM-derived Sca-1- and c-kit-positive cells in peripheral blood after ischemia was significantly lower in the HSF1-KO mice than in the WT mice. BM stem/progenitor cells from HSF1-KO mice showed a significant decrease in their recruitment to ischemic tissue and in migration, adhesion, and survival when compared with WT mice. Blood flow recovery in the ischemic hindlimb significantly decreased in WT mice receiving BM reconstitution with donor cells from HSF1-KO mice. Conversely, blood flow recovery in the ischemic hindlimb significantly increased in HSF1-KO mice receiving BM reconstitution with donor cells from WT mice. These findings suggest that HSF1 contributes to ischemia-induced angiogenesis by regulating the mobilization and recruitment of BM-derived stem/progenitor cells.

## Introduction

Angiogenesis in response to ischemia, including the sprouting of new capillary branches from pre-existing vessels, is an adaptive response in tissues with compromised blood supply, and it is important for perfusion after critical ischemia [1], [2], [3]. Recent evidence suggests that neovascularization within ischemic tissue involves the participation of bone marrow (BM)-derived stem/progenitor cells. The stem/progenitor cells in BM are mobilized into the peripheral blood in response to ischemia and are then recruited to the sites of ischemic injury to contribute to neovascularization, promoting blood flow recovery [4], [5], [6], [7]. Therefore, the mobilization and recruitment of BM-derived stem/progenitor cells are critical in ischemia-induced neovascularization. However, the exact cellular and molecular mechanisms regulating the mobilization and recruitment of these cells are fully not understood.

Heat shock factor 1 (HSF1) is an essential transcription factor in the response to cellular stress including a wide range of acute and chronic perturbation of pathophysiological states, and regulates the expression of heat shock proteins (HSPs) and numerous other molecules [8], [9], [10], [11]. HSF1 is also known to be induced and/or activated in response to hypoxia and ischemia [12]. However, the role of HSF1 in ischemia-induced angiogenesis remains unclear. Recent studies have shown that HSP90 or heme oxygenase-1 (HSP32), one of the target molecules of HSF1, may contribute to neovascularization after hindlimb ischemia [13], [14], [15]. Interestingly, it has been reported that heme oxygenase-1 is required for the mobilization of BM progenitor cells [15], [16]. Given that the expression of HSPs is mainly regulated by HSF1, it is possible that HSF1 regulates the mobilization and recruitment of BM-derived stem/progenitor and contributes, at least in part, to angiogenesis in response to ischemia.

In this study, we addressed the role of HSF1 in ischemia-induced angiogenesis. Using HSF1-knockout (KO) mice, we investigated the angiogenic response (blood flow recovery and microvessel density) and the mobilization and recruitment of BM-derived stem/progenitor cells after hindlimb ischemia.

## Materials and Methods

### Animals

The generation of HSF1-knockout (KO) mice on an ICR background has been described previously [17], [18], [19]. Wild-type (WT) mice of the same age, strain, and sex were used as controls. All mice used for these experiments were males aged 12 to 18 weeks. All animal procedures were approved by the Institutional Animal Care and Use Committee of Yamaguchi University and conformed to the Guide for the Care and Use of Laboratory Animals published by the US National Institutes of Health (NIH Publication No. 85-23, revised 1996).

### Ischemic hindlimb model

The mouse ischemic hindlimb model was created as described previously [20], [21], [22]. After mice were administered general anesthesia, the left femoral artery was exposed and ligated, and its branches were dissected free and excised.

### Measurement of blood flow in ischemic hindlimbs

Blood flow in the ischemic hindlimb was measured using a laser Doppler perfusion imaging system (PeriScan System, Perimed AB, Stockholm, Sweden), as described previously [20], [21], [22]. The recovery of perfusion in the ischemic hindlimb of each mouse was estimated by the percentage of limb blood flow, which was calculated by the average perfusion of the left hindlimb compared to that of the normal right hindlimb.

### Histological analysis of microvessel density

Mice were euthanized 21 days after induction of ischemia, and the quadriceps and adductor muscles were harvested and embedded in OCT compound (Sakura Finetechnical, Tokyo, Japan). To detect the development of microvessels in the ischemic muscles, frozen sections (5 µm) were stained for alkaline phosphatase using an indoxyl tetrazolium method, as described previously [20], [21]. A total of 15 different fields (×200-fold magnification) of three independent slides from different cross-sections were randomly selected for each mouse, and the number of microvessels and muscle fibers was counted. The density of microvessels was expressed as the microvessels/muscle fiber ratio.

### Measurement of angiogenic factors in plasma and limb muscle tissue

Plasma was obtained from the peripheral blood of mice both prior to and 3 days after limb ischemia. Tissue samples from ischemic and non-ischemic limbs were collected 3 days after ischemia. Protein extracts from the tissue samples were prepared by homogenization in a buffer containing 50 mM Tris-HCl, pH 7.2, 100 mM NaCl, 1% NP-40, and complete protease inhibitor mixture tablets (Roche, Mannheim, Germany). The concentration of vascular endothelial growth factor (VEGF) and stromal cell-derived factor-1 (SDF-1) in the plasma and limb tissue was determined using a commercially available ELISA kit (R & D Systems, Minneapolis, MN, USA) in accordance with the manufacturer's instructions.

### Analysis of mobilization of BM-derived stem/progenitor cells

To monitor the mobilization of BM-derived stem/progenitor cells, we collected peripheral blood both prior to and 3 days after limb ischemia. After density gradient centrifugation, peripheral blood mononuclear cells were stained with phycoerythrin (PE)-conjugated rat anti-mouse Sca-1 antibody (eBioscience Inc., San Diego, CA, USA) or c-kit antibody (eBioscience Inc.). Respective isotypes (eBioscience Inc.) were used as a negative control. Quantitative flow cytometric analysis was performed using a fluorescence-activated cell sorter (FACSCalibur; BD Biosciences, Franklin Lakes, NJ, USA). We analyzed the acquired data using CellQuest Pro software (BD Biosciences).

### Isolation of BM cells

BM was collected from the femur and tibia, and BM mononuclear cells were isolated by density gradient centrifugation, as described previously [20]. Isolated BM cells were used for subsequent analysis.

### Analysis of the amount of stem/progenitor cells in BM cells

To examine the amount of stem/progenitor cells in BM cells, Sca-1- and c-kit-positive cells in freshly isolated BM cells from WT or HSF1-KO mice were measured by flow cytometry, as described above.

### Assessment of recruitment of BM cells into limb tissue

To investigate the capacity for recruitment of BM cells into ischemic tissue, isolated BM cells from WT or HSF1-KO mice were labeled with intracellular fluorescent dye of carboxyfluorescein diacetate succinimidyl ester (CFSE, Molecular Probes, Inc., Eugene, OR, USA) as described previously [22]. The CFSE-labeled BM cells (8×10^6^) were intravenously injected into WT mice 1 day after induction of limb ischemia. Mice were euthanized 1 day after the injection of BM cells, and the muscles from the ischemic hindlimbs were harvested. Frozen sections were used to evaluate the recruitment in ischemic tissue by direct vision of CFSE-positive cells under a fluorescence microscope (×200-fold magnification). Nuclei were stained with 4′,6-diamidine-2′-phenylindole (DAPI). A total of 15 different fields from three independent slides for different cross-sections were randomly selected for each mouse, and the number of CFSE-positive cells was counted. The results are expressed as the number of CFSE-positive cells/field.

### Migration assay

Cell migration was determined by a modified Boyden chamber assay [23]. Briefly, isolated BM cells (2×10^5^) were suspended in 100 µl of serum-free RPMI 1640 containing 0.05% bovine serum albumin (BSA) and were added to the upper chamber (Transmembrane, 8 µm pore size, Corning Incorporated, Corning, NY, USA). The lower chamber of the apparatus was filled with 600 µl of serum-free RPMI 1640 containing 0.05% BSA without or with recombinant mouse SDF-1 (100 ng/ml, R&D systems). The whole apparatus was incubated at 37°C for 6 hours. The number of cells migrating to the lower chamber was counted in four random microscopic fields (×200-fold magnification). Data are expressed as the number of migrated cells/field.

### Adhesion assay

The cell adhesion assay was performed as described previously [22]. Isolated BM cells suspended in RPMI 1640 containing 10% FBS were seeded on fibronectin-coated 24-well plates (1×10^6^/ml/well) and incubated at 37°C in 5% CO_2_. After 1 day of culture, non-adherent cells were removed by tapping and gently washing the wells three times with PBS. The number of adherent cells was counted in five random microscopic fields (×200-fold magnification) in each well. Data are expressed as the number of adherent cells/field.

### Survival analysis

Cell viability was determined by trypan blue dye exclusion, as described previously [20], [21]. Isolated BM cells suspended in RPMI 1640 containing 10% FBS. Cells were seeded on 96-well culture plates (2×10^5^/200 µl/well) and were then incubated at 37°C in 5% CO_2_. After 1 day of culture, the cell survival rate was calculated as the percentage of surviving cells among all of the seeded cells.

### BM reconstitution and blood flow recovery of ischemic limbs

To assess the contribution of BM-derived cells to ischemia-induced angiogenesis, BM transplantation (BMT) was performed, as previously described [24]. Briefly, WT or HSF1-KO mice were subjected to lethal irradiation (10 Gy) and were then implanted intravenously with 8×10^6^ BM cells that had been isolated from WT or HSF1-KO mice. We made the following 4 groups of chimeric mice: recipient WT mice implanted with donor BM cells from WT mice; recipient WT mice implanted with donor BM cells from HSF1-KO mice; recipient HSF1-KO mice implanted with donor BM cells from WT mice; and recipient HSF1-KO mice implanted with donor BM cells from HSF1-KO mice.

The ischemic hindlimb model was implemented in these chimera mice 8 weeks after BMT, and blood flow in the ischemic hindlimb was measured using a laser Doppler perfusion imaging system (PeriScan System) 14 days after ischemia, as described above.

### Analysis of HSF1 expression in limb tissue and BM cells after ischemia

To examine the expression of HSF1 in limb tissues and BM cells in WT mice after hindlimb ischemia, we performed western blot analysis, as described previously [22], [25] . Tissue samples from ischemic and non-ischemic limbs were collected 3 days after ischemia, and protein extracts were prepared as described above. BM cells were collected both prior to and 3 days after hindlimb ischemia, and lysed in a buffer containing 20 mM Tris-HCl, pH 7.5, 150 mM NaCl, 1% Triton X-100, and complete protease inhibitor mixture tablets (Roche). Equal amount of protein from tissue samples or cell lysates was subjected to sodium dodecyl sulfate-polyacrylamide gel electrophoresis, and transferred onto a polyvinylidene difluoride membrane (Millipore, Bedford, MA, USA). The blotted membranes were incubated with primary antibodies to HSF1 (10H8; Enzo Life Science, Inc., Farmingdale, NY, USA) or β-actin (Sigma, St. Louis, MO, USA), as an internal control. Corresponding horseradish peroxidase-conjugated antibodies were used as the second antibody. Signals were visualized with an enhanced chemiluminescence Western blot detection system (GE Healthcare UK Ltd, Buckinghamshire, United Kingdom) and recorded with luminescent image analyzer (LAS-1000; Fuji Film Co., Tokyo, Japan). Each band was quantified using Image J software, and level of HSF1 was normalized to those of β-actin. Data are expressed as the fold increase.

### Statistical analysis

All data are expressed as the mean ± SD. Differences between mean values of multiple groups were evaluated using ANOVA followed by Scheffe's procedure. Comparisons between two groups were made using unpaired Student's *t*-tests. A value of P<0.05 was considered significant. All analyses were performed with StatView software (Version 5.0).

## Results

### Impaired ischemia-induced angiogenesis in HSF1-KO mice

**Figure 1:**
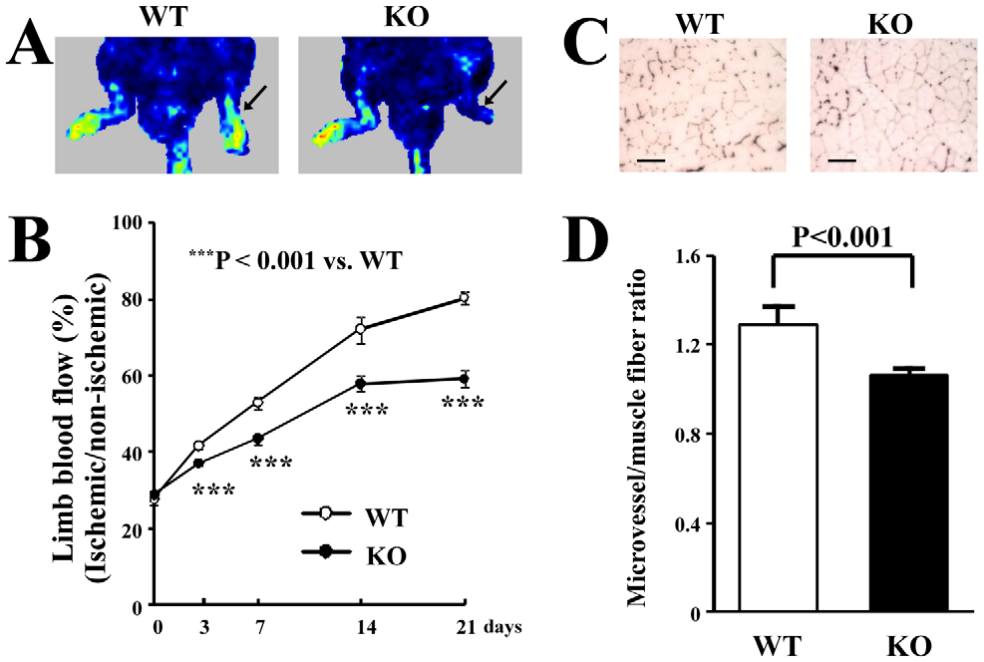
Angiogenic response in ischemic limbs. (A) Representative color-coded images representing blood flow distribution. Arrows indicate the ischemic hindlimbs. (B) Quantitative analysis of the time course of recovery of blood flow in ischemic limbs. (C) Representative images of microvessels stained for alkaline phosphatase. Bars indicate 100 µm. (D) Quantitative analysis of microvessel density. Quantitative analysis revealed that the blood flow (B) and microvessel density (D) in the ischemic hindlimb significantly decreased in the HSF1-KO mice compared to the WT mice, 21 days after the induction of ischemia (n = 5 or 6 animals/group).

We examined the angiogenic response in ischemic limbs in the HSF1-KO mice. We took serial measurements of the perfusion of the ischemic hindlimbs prior to, and 3, 7, 14, and 21 days after ischemia. Laser Doppler perfusion imaging showed profound differences in blood flow in the ischemic hindlimbs between HSF1-KO and WT mice at 21 days (Figure 1A). Quantitative analysis also showed that the percentage of limb blood flow was significantly lower in the HSF1-KO mice than in WT mice (P<0.001) (Figure 1B). To detect microvessels in the ischemic muscles, staining for alkaline phosphatase was performed 21 days after ischemia. Histological analysis revealed that microvessel density was significantly lower in the HSF1-KO mice than in WT mice (P<0.001) (Figure 1C and 1D). All of these findings indicate that the HSF1-KO mice showed an impaired angiogenic response in the ischemic limbs.

### VEGF and SDF-1 levels in plasma and limb tissue of HSF1-KO mice

**Figure 2:**
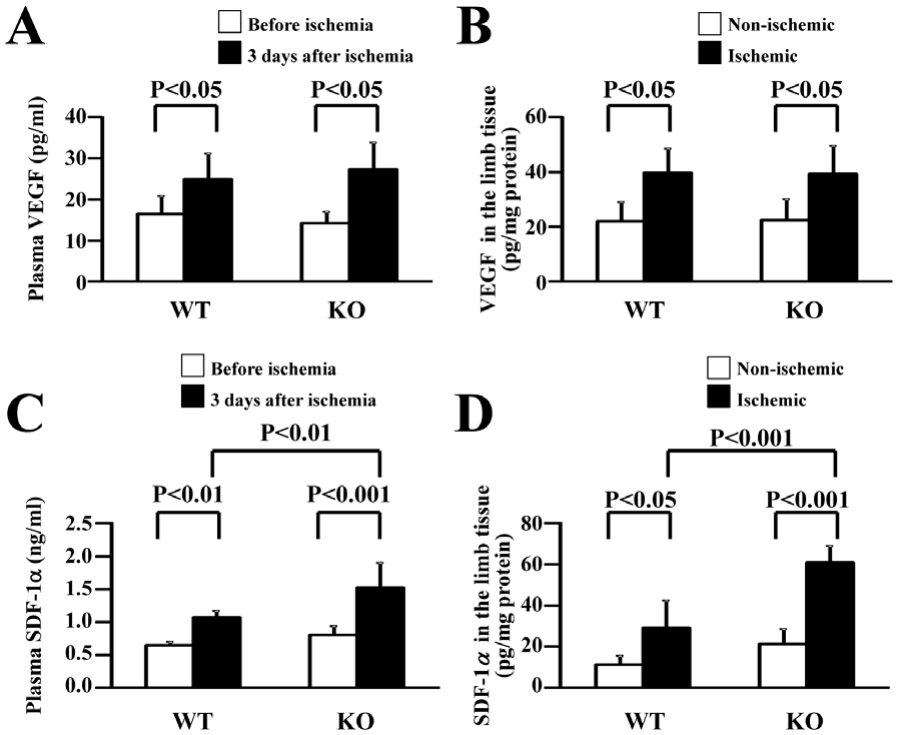
VEGF and SDF-1 levels in plasma and limb tissue. ELISA analysis showed that VEGF (A, B) and SDF-1 (C, D) levels in the plasma (A, C) and ischemic limb tissue (B, D) increased in both HSF1-KO and WT mice, 3 days after ischemia (n = 5–8 animals/group).

To investigate the reason for the impaired angiogenesis in the HSF1-KO mice, we examined how the levels of VEGF and SDF-1, two well-known critical factors regulating angiogenesis [26], [27], [28], [29], in the plasma and limb tissue, differed between KO and WT mice in response to ischemia. We found that the VEGF levels in the plasma and limb tissue in the HSF1-KO mice increased after inducing limb ischemia (Figure 2A and 2B), and that VEGF levels in the plasma and ischemic tissue did not significantly differ between HSF1-KO and WT mice 3 days after ischemia. We also observed increased levels of SDF-1 in the plasma and limb tissue in the HSF1-KO mice after ischemia, which was similar to WT mice (Figure 2C and 2D). These results suggest that impaired angiogenesis in the HSF1-KO mice likely did not result from decreased levels of VEGF and SDF-1.

### Decreased mobilization of BM-derived stem/progenitor cells in HSF1-KO mice

**Figure 3:**
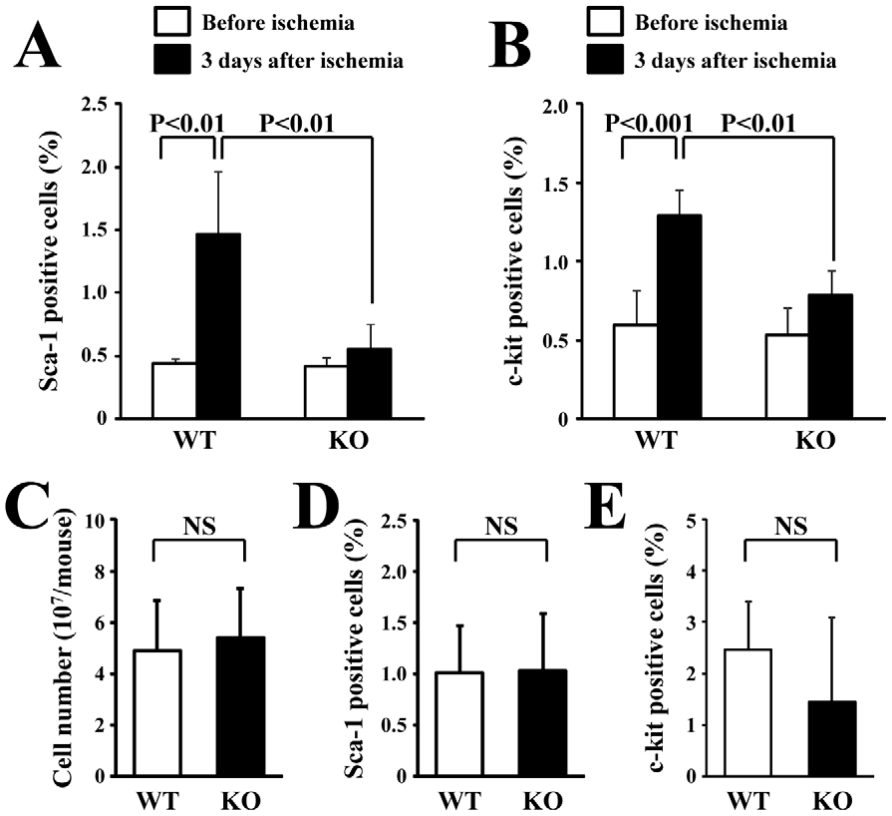
Mobilization of BM-derived cells in the peripheral blood. Flow cytometric analysis showed that the mobilization of BM-derived Sca-1- (A) and c-kit-positive cells (B) in the peripheral blood significantly increased in the WT mice but not in the HSF1-KO mice, 3 days after ischemia (n = 4 animals/group). However, the total amount (C) or the subpopulation of Sca-1- (D) and c-kit-positive cells (E) in BM cells did not differ between WT and HSF1-KO mice (n = 3 animals/group).

To evaluate the mobilization of BM stem/progenitor cells, we examined the mobilized these cells in response to ischemia. Flow cytometric analysis showed that the Sca-1- and c-kit-positive cells in the peripheral blood significantly increased in the WT mice but not in the HSF1-KO mice 3 days after ischemia (Figure 3A and 3B). Sca-1- and c-kit-positive cells in the peripheral blood before ischemia did not significantly differ between WT and HSF1-KO mice. Furthermore, there was no significant difference in the total amount (Figure 3C) or the amount of cells positive for Sca-1 (Figure 3D) and c-kit (Figure 3E) between BM cells from WT and HSF1-KO mice. These results indicate a decreased mobilization of BM-derived stem/progenitor cells in the HSF1-KO mice, although the amount of stem/progenitor cells in BM cells did not differ between WT and HSF1-KO mice.

### Impaired recruitment of BM cells from HSF1-KO mice to ischemic tissue

**Figure 4:**
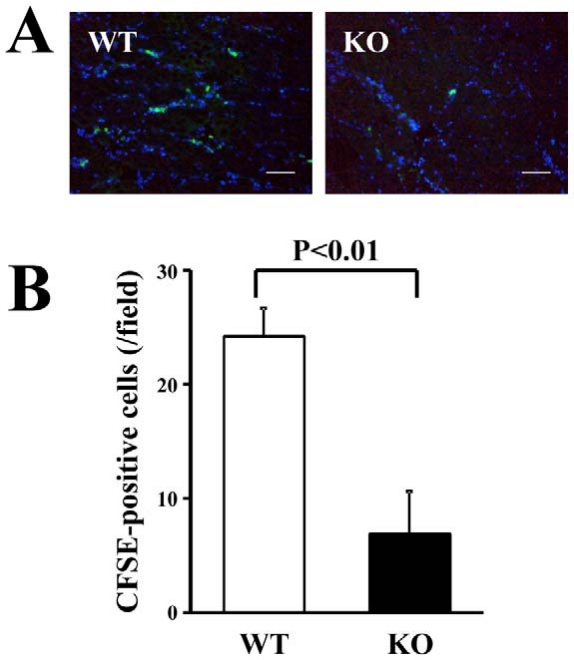
Recruitment of BM cells to ischemic limbs. (A) CFSE-positive cells (green) recruited to the ischemic limbs were visualized directly under a fluorescent microscope. Nuclei were stained with DAPI (blue). Bars indicate 100 µm. (B) Quantitative analysis of CFSE-positive cells showed a significant decrease in the recruitment of the BM cells from KO mice, compared with those from WT mice (n = 3 animals/group).

We next examined the capacity for recruitment of BM cells from WT or HSF1-KO mice to ischemic tissues of WT mice. Despite the intravenous injection of the same number of CFSE-labeled BM cells, the accumulation of BM cells from the HSF1-KO mice was remarkably lower in the ischemic tissue than in those from the WT mice (Figure 4A). Quantitative analysis showed that the number of CFSE-labeled cells in the ischemic tissue was significantly lower in the BM cells from the HSF1-KO mice than in those from the WT mice (P<0.01) (Figure 4B). These data suggest that the recruitment of BM cells to the ischemic tissue was impaired in the HSF1-KO mice.

### Functional impairment of BM cells from HSF1-KO mice

**Figure 5:**
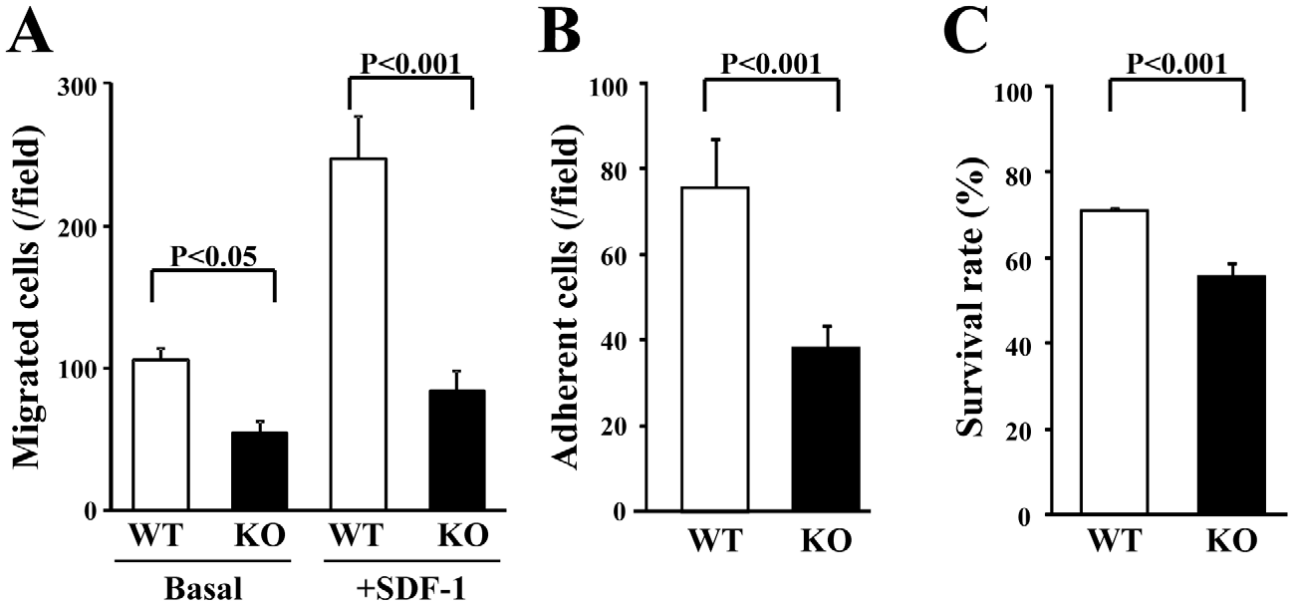
*In vitro* assessment of BM cell function. (A) Cell migration was determined by a Boyden chamber assay. (B) Cells were seeded on fibronectin-coated plates, and the number of adherent cells was counted after 1 day of culture. (C) Cell viability was determined by trypan blue dye exclusion after 1 day of culture. Quantitative analysis revealed that migration (A), adhesion (B), and survival (C) were all impaired in the BM cells from KO mice, compared with those from WT mice (n = 3 animals/group).

In addition, we performed an *in vitro* assessment of BM cell function, including migration, adhesion, and survival, because these functions are important for the *in vivo* mobilization and recruitment of BM-derived cells [5], [6], [7]. The number of migrated cells was significantly lower in the HSF1-KO mice than in the WT mice, in both absence and presence of SDF-1 (P<0.05 and P<0.001, respectively) (Figure 5A). The number of adherent cells significantly decreased in the HSF1-KO mice than in the WT mice (P<0.001) (Figure 5B). The survival rate was significantly lower in the HSF1-KO mice than in the WT mice (P<0.001) (Figure 5C). These results suggest that a deficiency of HSF1 contributes to the impairment of the function of BM cells in survival, adhesion, and movement.

### Blood flow recovery in ischemic limbs after BMT

**Figure 6:**
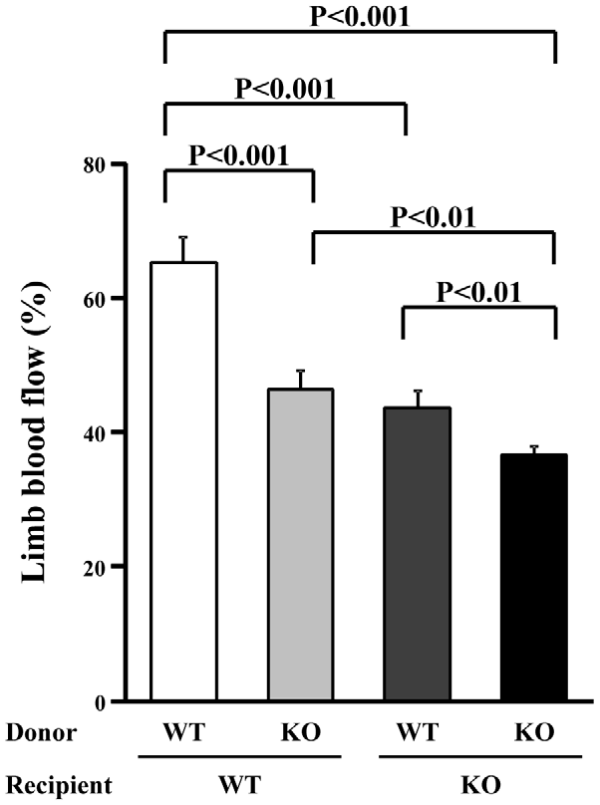
Blood flow in ischemic limbs after BM reconstitution. The blood flow of the ischemic hindlimbs of the chimeric mice after mismatched BMT was measured by laser Doppler perfusion imaging. The recovery of blood flow in the ischemic hindlimb significantly decreased in recipient WT mice receiving BM reconstitution with donor cells from KO mice. Conversely, the blood flow in the ischemic hindlimb significantly improved in recipient KO mice receiving BM reconstitution with donor cells from WT mice (n = 5 animals/group).

To further confirm our hypothesis that HSF1 contributes to ischemia-induced angiogenesis by regulating the mobilization and recruitment of BM-derived cells, we induced hindlimb ischemia in the chimeric mice after mismatched BMT. The recovery of blood flow in the ischemic hindlimb significantly decreased in recipient WT mice receiving BM reconstitution with donor BM cells from HSF1-KO mice (P<0.001). Conversely, the blood flow in the ischemic hindlimb significantly increased in recipient HSF1-KO mice receiving BM reconstitution with donor BM cells from WT mice (P<0.01), but it was not as much as that of the recipient WT mice receiving BM reconstitution with donor BM cells from WT mice (P<0.001) (Figure 6). All of these findings suggest that, in the HSF1-KO mice, impaired neovascularization in response to ischemia might be attributable, at least in part, to the dysfunction of BM cells.

### Increased expression of HSF1 in ischemic limb tissues and BM cells after ischemia

**Figure 7:**
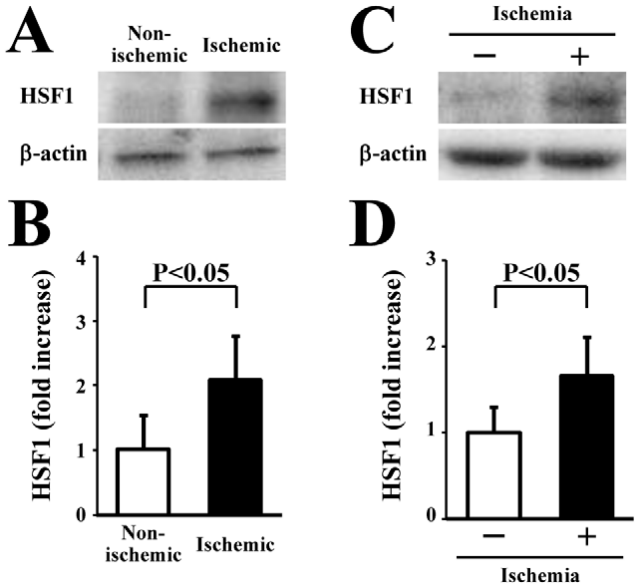
HSF1 expression in limb tissue and BM cells in response to ischemia. Representative images of western blot analysis for HSF1 in the limb tissue (A) and BM cells (C). Quantification after normalization to β-actin showed that HSF1 expression was upregulated in ischemic tissue (B) and BM cells (D) after the induction of ischemia (n = 4 animals/group).

We investigated the change in the expression of HSF1 protein in limb tissues and BM cells in WT mice after limb ischemia. Western blot analysis showed that the expression of HSF1 was significantly higher in the ischemic limb tissue than in the non-ischemic limb tissue (P<0.05) (Figure 7A and 7B). Moreover, HSF1 expression was also significantly increased in the BM cells from mice with the induction of limb ischemia when compared to the BM cells from healthy mice (P<0.05) (Figure 7C and 7D). These results suggest that the induction of limb ischemia can induce the expression of HSF1 in the local ischemic limb tissue and BM cells.

## Discussion

The novel finding of this study is that a deficiency in HSF1 results in an impairment in neovascularization after ischemia. Our study provides the first evidence that HSF1 plays a pivotal role in ischemia-induced neovascularization by regulating the mobilization and recruitment of BM-derived stem/progenitor cells.

We found that HSF1-KO mice have impaired ischemia-induced neovascularization, as shown by the lower microvessel density and blood flow in the ischemic limbs. To elucidate the reason for the reduced angiogenic potential in the HSF1-KO mice, we examined the VEGF and SDF-1 in the plasma and ischemic tissue, as VEGF and SDF-1 are known to be induced by ischemia/hypoxia and contribute to angiogenesis [26], [27], [28], [29]. However, the fact that VEGF and SDF-1 levels in the plasma and ischemic tissue increased, even in the HSF1-KO mice after induction of ischemia that was similar to the WT mice, indicated that the impairment in angiogenic potential in the HSF1-KO mice is likely not attributable to decreased levels of VEGF and SDF-1. Although VEGF and SDF-1 could be partly secreted by the recruited BM-derived cells in the ischemic limb, ischemia is also known to induce the production of VEGF and SDF-1 from various types of cells, including the endothelial cells and myoblasts. Therefore, it was not surprising that these host cells, rather than the recruited BM cells, might contribute to release the most amount of VEGF within the limb tissue after the induction of ischemia.

Next, we investigated the mobilization and recruitment of BM stem/progenitor cells, as BM-derived stem/progenitor cells have been shown to contribute to ischemia-induced neovascularization [4], [5], [6], [7]. The mobilization of BM-derived stem/progenitor cells was blunted in HSF1-KO mice after the induction of ischemia, and the recruitment of BM cells from HSF1-KO mice into ischemic tissue also decreased. Furthermore, BM stem/progenitor cells from HSF1-KO mice had decreased migration, adhesion, and survival *in vitro*. Considering that the mobilization and recruitment of BM-derived stem/progenitor cells are mediated by migration, adhesion, and survival [5], [6], [7], it is likely that the functional impairment of these cells is responsible for their defective mobilization and recruitment. Taken together, we may attribute the impaired neovascularization in the response to ischemia in the HSF1-KO mice to the defective mobilization, recruitment, and function of BM-derived stem/progenitor cells. Indeed, we demonstrated that blood flow in the ischemic hindlimb significantly decreased in WT mice receiving BM reconstitution with cells from HSF1-KO mice.

It has been shown that the mobilization, recruitment, and function in BM stem/progenitor cells are impaired in patients with advanced age and systemic diseases, such as diabetes [5], [30], [31]. Therefore, it will be interesting for future studies to examine whether the defective mobilization, recruitment, and function of BM stem/progenitor cells in these patients are related to the inactivation and decreased expression of HSF1.

Here, we have showed that the defective mobilization and recruitment of BM-derived stem/progenitor cells are related to the impairment of ischemia-induced angiogenesis. However, defective mobilization and recruitment of these cells do not fully account for the impaired neovascularization in the response to ischemia in HSF1-KO mice, because the blood flow in the ischemic hindlimb was lower in the HSF1-KO mice receiving WT BM reconstitution than in the WT mice receiving WT BM reconstitution. Considering that recent studies have revealed the contribution of HSP90 or heme oxygenase-1 to neovascularization [13], [14], [15], there is a possibility that changes in HSP expression within limb tissue in response to ischemia may in part be associated with the reduced angiogenic potential in the HSF1-KO mice. Interestingly, we found the increased expression of HSF1 in the limb tissue after ischemia. It keeps unclear how the enhanced HSF1 effects the expression of HSPs, that are known to play a role of angiogenesis in response to ischemia. Moreover, it remains to be seen whether HSF1 affects the function of other types of cells, including endothelial cells and myoblasts, for angiogenesis within the limb tissue after the induction of ischemia. Further experiments are also required to uncover whether HSF1 could contribute to the engraftment efficiency of BMT. All of these will help us to elucidate the additional roles of HSF1 in ischemia-induced angiogenesis.

In conclusion, this study showed that ischemia-induced neovascularization is impaired in HSF1 KO mice, which is likely related to decreased mobilization and recruitment of BM-derived stem/progenitor cells. Although further experiments are required to examine in detail how HSF1 affects the number and function of BM-derived stem/progenitor cells, HSF1 likely plays a role in ischemia-induced angiogenesis by regulating the mobilization and recruitment of BM stem/progenitor cells. HSF1 is expected to be a new therapeutic target for the treatment of ischemic diseases.
